# Diagnostic predictive value of platelet indices for discriminating hypo productive versus immune thrombocytopenia purpura in patients attending a tertiary care teaching hospital in Addis Ababa, Ethiopia

**DOI:** 10.1186/s12878-016-0057-5

**Published:** 2016-07-01

**Authors:** Mikias Negash, Aster Tsegaye, Amha G/Medhin

**Affiliations:** College of Health Science, Department of Medical Laboratory Science, Addis Ababa University, Addis Ababa, Ethiopia; Department of Internal Medicine, Addis Ababa University, Addis Ababa, Ethiopia

**Keywords:** Bone marrow, Platelet indices, Immune thrombocytopenia purpura (hyperdestructive thrombocytopenia), Hypoproductive thrombocytopenia

## Abstract

**Background:**

Bone marrow examination may be required to discriminate causes of thrombocytopenia as hypoproductive or hyperdestructive. However, this procedure is invasive and time consuming. This study assessed the diagnostic value of Mean Platelet Volume (MPV), Platelet Distribution Width (PDW) and Platelet Large Cell-Ratio (P-LCR) in discriminating causes of thrombocytopenia as hypoproductive or hyperdestructive (Immune thrombocytopenia purpura).

**Method:**

A prospective cross-sectional study was conducted on 83 thrombocytopenic patients (Plt < 150 × 10^9^/L). From these, 50 patients had hypoproductive and the rest 33 Immune Thrombocytopenia Purpura (ITP). Age and sex matched 42 healthy controls were included as a comparative group. Hematological analysis was carried out using Sysmex XT 2000i 5 part diff analyzer. SPSS Version16 was used for data analysis. A two by two table and receiver operating characteristic (ROC) curve was used to calculate sensitivity, specificity, positive and negative predictive values, for a given platelet indices (MPV, PDW and P-LCR). Student *t* test and Mann Whitney *U* test were used to compare means and medians, respectively. Correlation test was used to determine associations between continuous variables.

**Results:**

All Platelet indices were significantly higher in ITP patients (*n* = 33) than in hypoproductive thrombocytopenic patients (*n* = 50) (*p* < 0.0001). In particular MPV and P-LCR have larger area under ROC curve (0.876 and 0.816, respectively), indicating a better predictive capacity, sensitivity and specificity in discriminating the two causes of thrombocytopenia. The indices were still significantly higher in ITP patients compared to 42 healthy controls (*p* < 0.0001). A significant negative correlation was observed between platelet count and platelet indices in ITP patients, (*p* < 0.001).

**Conclusion:**

MPV, PDW and P-LCR help in predicting thrombocytopenic patients as having ITP or hypoproductive thrombocytopenia. If these indices are used in line with other laboratory and clinical information, they may help in delaying/ avoiding unnecessary bone marrow aspiration in ITP patients or supplement a request for bone morrow aspiration or biopsy in hypoproductive thrombocytopenic patients.

## Background

Platelets play a pivotal role in the first steps of clot formation by adhering to damaged blood vessel as well as donating their membrane phospholipids for the activation of coagulation factors [1]. A platelet count of less than 150 × 10^9^/L is considered to be thrombocytopenic. The two main causes of thrombocytopenia excluding pseudo thrombocytopenia are increase destruction or peripheral consumption (hyper-destructive thrombocytopenia), such as immune thrombocytopenic purpura (ITP), disseminated intravascular coagulation (DIC), and thrombotic thrombocytopenic purpura (TTP). Whereas decreased platelet productions (hypo-production thrombocytopenia) are associated with a number of bone marrow diseases [2].

The gold standard method for discriminating these two causes is bone marrow examination. No consensus is reached regarding the necessity of a bone marrow examination in the evaluation of idiopathic thrombocytopenic purpura. Due to its invasiveness and being unfriendly for the patients, this procedure is not recommended as first line diagnosis. ITP still remains a diagnosis by exclusion due to lack of accurate clinical and laboratory parameters [3].

Studies conducted elsewhere showed that platelet indices like Mean Platelet Volume (MPV) are sensitive, specific and have diagnostic predictive value in discriminating ITP (hyper-destructive thrombocytopenia) from hypoproductive [4–7]. However, many of these studies either evaluated a single parameter like MPV or compared patients with one type of disease category. These platelet indices such as MPV (average size of platelet), Platelet Distribution Width (PDW; the width measured at 20 % of the platelet histogram) and Platelet Large Cell-Ratio (P-LCR; percentage of platelets with size more than 12 fl) are usually available as part of hematology outputs of many of the automated analyzers. A study in Ethiopia reported that majority of the hematological parameters including platelet indices are underutilized in clinical patient management [8]. This hospital based prospective study was conducted to demonstrate the discriminating potential of the three platelet indices between Immune thrombocytopenia purpura versus hypoproductive thrombocytopenia and determine a cut of value with the highest prediction in Ethiopian patients.

## Methods

### Study site

The study was conducted at Tikur Anbessa Specialized Teaching Hospital of Addis Ababa University, Ethiopia. This is a pioneer tertiary hospital which manages patients with hematological disorders in our country. Suspected or confirmed hematological patients with platelet count below150 x 10^9^/L who volunteered to participate were enrolled in to the study.

### Laboratory analysis

Blood sample for complete blood count (CBC) analysis was collected into 5 ml EDTA anti-coagulated tubes. CBC analysis was performed using Sysmex XT 2000i fully automated hematology analyzer (Kobe, Japan) based on the manufacturer’s protocol. All blood samples were analyzed in less than 4 h of blood collection. This analyzer measures white blood cells (WBC) and reticulocytes with an optical detector block based on the flow cytometry principle, red blood cells (RBC) and platelet counts are analyzed via the impedance method. The performance of the instrument was monitored by running quality control materials. A peripheral blood smear was also reviewed to estimate platelet counts, rule out pseudo thrombocytopenia and fragments of cells like shistocytes.

### Study participants

Based on clinical and laboratory information, including preceding and follow-up laboratory data, the patients were divided into two groups; those with marrow disease (hypoproductive) and those with ITP (hyperdestructive). Suspected and confirmed hematological patients with platelet counts below 150 × 10^9^/L were traced from wards and follow up hematology clinic. Patients’ medical records were reviewed and any newly diagnosed patients were followed to find out the cause of their thrombocytopenia. The presence of bone marrow disease was diagnosed based on bone marrow examination and it was performed by residents and senior pathologists at Tikur Anbessa pathology laboratory. In this study 83 thrombocytopenic patients were enrolled; 50 had hypoproductive thrombocytopenia while the rest 33 were ITP patients. Bone marrow examination was performed in all 50 hypoproductive patients and in 10 of the 33 ITP patients as part of their medical follow up and diagnosis. ITP Patients who did not have bone marrow examination were monitored during their follow up period to ascertain the diagnosis (for a minimum of 6–8 months). Patients living with the human immunodeficiency virus (HIV) and who had been transfused with platelet concentrate or whole blood in the previous 9 days were excluded from the study. As a control group, 42 age and sex matched apparently healthy individuals were included. Ethical approval was obtained from department of medical laboratory and department of internal medicine of Addis Ababa University. Verbal consent was obtained from adults and parents/guardians of children. In addition assent was secured from children aged 12–17 years provided their parents/guardians consented for their participation.

### Statistical analysis

The data was entered and analyzed using SPSS Version 16 (SPSS INC, Chicago, IL, USA). A two by two table and receiver operating characteristic (ROC) curve was used to calculate sensitivity, specificity, positive predictive value (PPV), negative predictive value (NPV), for a given platelet indices (MPV, PDW and P-LCR). Student *t* test and Mann Whitney *U* test were used to compare means and medians, respectively; Correlation test was used to see the association between continuous variables. A *P*-value of < 0.05 was considered as statistically significant.

## Result

A total of 83 patients were enrolled in this study. Among these patients, 50 had hypoproductive thrombocytopenia (bone marrow disease) and 33 were ITP patients. The mean age (± standard deviation) of the patients was 41 ± 17.8 and 27 ± 16.3) years, respectively. Females were the predominant study participants in ITP patients, while males were predominant in the hypo productive group (*p* = 0.008). For comparison, 42 apparently healthy controls were included. This is summarized in Table 1.

**Table 1:** Socio-demographic characteristics of patients and apparently healthy controls

Variable	Number	Sex		Age in years Mean (SD)
		Male	Female	
Hypoproductive	50	33	17	41 (17.8)
ITP	33	12	21	27 (16.3)
Healthy controls	42	26	16	25 (6.8)
Total	125	71	54	

### Comparison of platelet count and Platelet indices between study participants

The platelet count and the platelet indices were compared between ITP, hypoproductive patients and healthy controls. The platelet count was not significantly different between hypoproductive and ITP patients. However, all the platelet indices MPV, PDW and P-LCR were significantly higher (*P* < 0.0001) in patients with ITP than in patients with hypoproductive thrombocytopenia. All the platelet indices were significantly higher in ITP patients as compared to healthy controls (Table 2), but no such significant difference was observed between the healthy controls and patients with hypo-productive thrombocytopenia except for their MPV (*P* = 0.01).

**Table 2:**
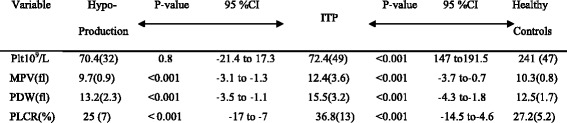
Comparison of mean platelet count and mean platelet indices between hypo-productive, ITP patients and healthy controls

*MPV* mean platelet volume, *PDW* platelet distribution width, *P-LCR* platelet larger cell ratio; results are presented as mean, values in bracket are standard deviation; *P* value was considered significant at level of 0.05

### Thrombocytopenia causes

The different causes of thrombocytopenia were analyzed for the two patient groups along with the respective platelet count and platelet indices. The main cause of thrombocytopenia in hypoproductive patients was bone marrow toxicity secondary to chronic myelogenous leukemia (CML) chemotherapy (Table 3). In patients with ITP, the predominant cause was chronic ITP which accounted 28 out of the 33 cases. The remaining acute ITP patients had an average low platelet count of 12.6 × 10^9^/L with an MPV of 16.6 fl, PDW 19 fl and P-LCR 51.5 %.

**Table 3:** Causes of thrombocytopenia in hypoproductive patients and respective average platelet count and platelet indices

Diagnosed causes of thrombocytopenia	Number	Pltx10^9^/L	MPV (fl)	PDW (fl)	P-LCR (%)
BM toxicity secondary to CML chemotherapy	27	72	9.5	12.3	23
ALL on chemotherapy	5	57	10	13.7	27.5
ALL chemotherapy naïve	2	12	11	13	34
Myelodysplastic syndrome	1	88	8.8	15.3	17
AML on chemotherapy	3	77	8.5	14.4	18
Myelofibrosis	2	44.5	9.5	16.5	24
Erythroid hyperplasia	2	77	10.4	14	31
CLL chemotherapy naïve	4	79	10.4	16.2	34
BM toxicity to lymphoma chemotherapy	4	56	9.9	13	25
Total/average	50	70.4	9.7	13.2	25

*CML* chronic myelogenous leukemia, *BM* bone marrow, *ALL* acute lymphocytic leukemia, *AML* Acute myeloid leukemia, *CLL* chronic lymphocytic leukemia

### Correlation between platelet parameters

**Fig. 1:**
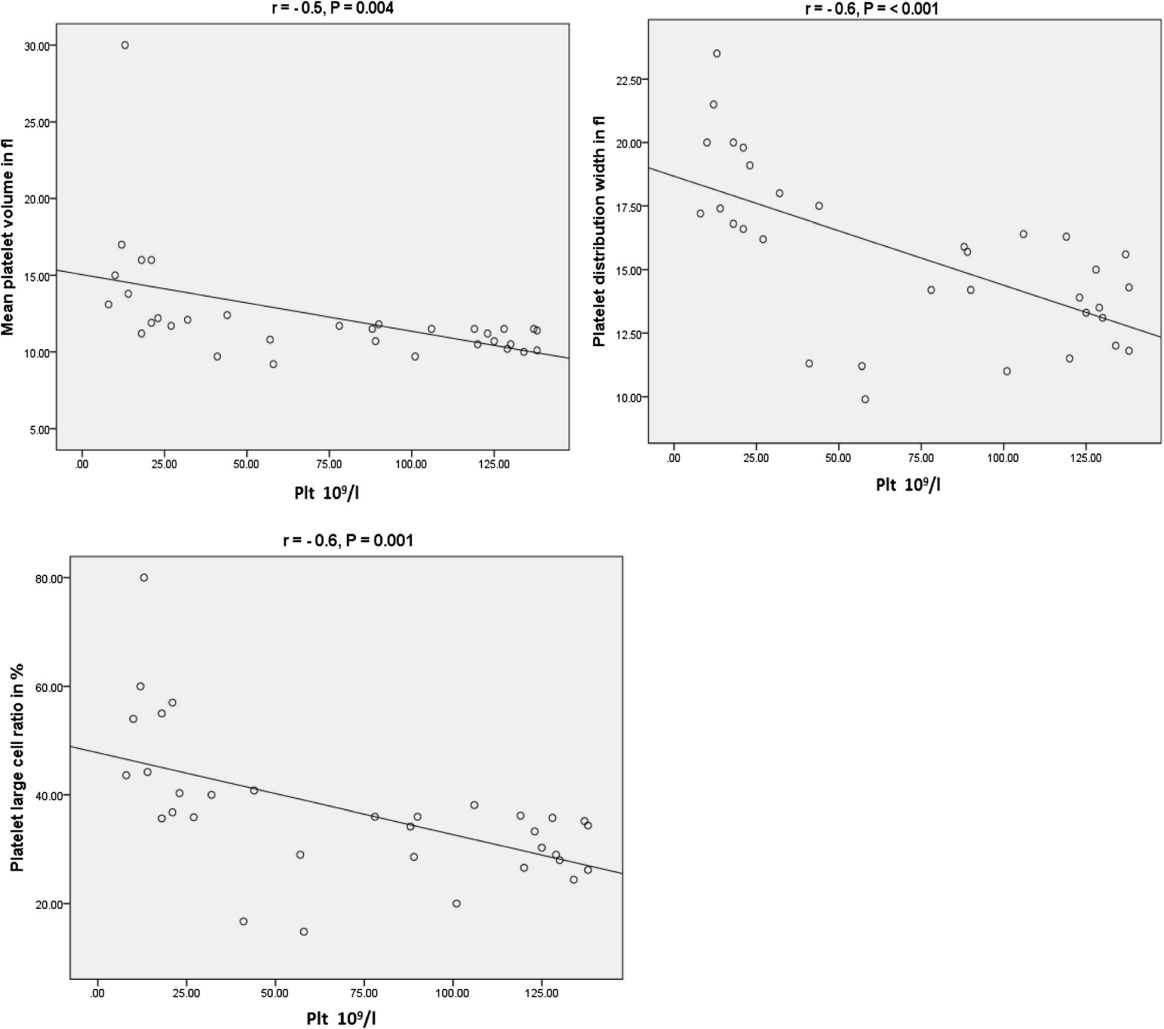
Correlation between platelet count and MPV, PDW, P-LCR in patients with ITP *r* = correlation coefficient

There was statistically significant negative correlation between platelet count and the platelet indices in ITP patients (Fig. 1). However, the platelet count and platelet indices did not show significant correlation in Hypoproductive patients (the correlation coefficient between the platelet count and MPV, PDW, and P-LCR was 0.09, -0.13 and -0.01 respectively). A significant negative correlation between the platelet count and the indices was observed in the 42 healthy controls with a correlation coefficient of -0.38, -0.37 and -0.39 for MPV, PDW, and P-LCR respectively.

### ROC Curve for analysis of sensitivity and specificity

**Fig. 2:**
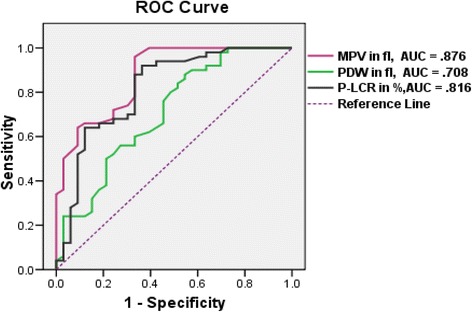
ROC curve comparing area covered by MPV, PDW, P-LCR to identify hypoproductive cases among thrombocytopenic patients

The area under the curve (AUC) gives the probability that a patient with bone marrow disease has lower values of the measurement (here MPV, PDW and P-LCR). The AUC in Fig. 2 shows lines shifting towards the left upper corner particularly for MPV and P-LCR giving an area of .876 (87.6 %) and .816 (81.6 %), respectively.

Sensitivity and specificity were extrapolated from different coordinate points of the ROC curve. The platelet indices, MPV and P-LCR in particular have better sensitivity, specificity and predicative value in discriminating the two types of thrombocytopenia (Tables 4 and 5). An MPV of <10.75 fl can identify thrombocytopenic patients as hypoproductive with 74 % sensitivity, 70 % specificity, 79 % PPV and 64 % NPV. These indices can also identify patients with ITP, such that an MPV of >11.05 fl can identify patients as having ITP with 67 % sensitivity, 95 % specificity, 88 % PPV and 81 % NPV.

**Table 4:** Sensitivity and specificity of the platelet indices for diagnosis of hypoproductive thrombocytopenia (bone marrow disease) at different cut off points from ROC curve coordinate

MPV (fl)	Sensitivity %	Specificity %	P-LCR (%)	Sensitivity %	Specificity %
<10.75	74	70	<31.4	76	67
<10.95	90	67	<33.15	88	67
<11.05	95	67	<33.7	90	64
<11.3	100	61	<34.3	92	61
PDW (fl)					
<15.5	76	55			
<15.65	80	52			
<15.75	82	49			
<15.85

**Table 5:** Sensitivity and specificity of the platelet indices for diagnosis of ITP at different cut off points from ROC curve coordinates

MPV (fl)	Sensitivity %	Specificity %	P-LCR (%)	Sensitivity %	Specificity %
>11.05	67	95	>33.15	67	88
>10.75	70	74	>30.05	70	70
>10.15	85	66	>28	82	66
>9.95	91	64	>26.15	88	64
PDW (fl)					
>14.25	61	62			
>14.1	67	40			
>13.05	79	50			

*MPV* mean platelet volume, *PDW* platelet distribution width, *P-LCR* platelet larger cell ratio, *ROC* receiver operating characteristics curve, *ITP* immune thrombocytopenia purpura

## Discussion

Simple, inexpensive and non invasive tests like MPV have been reported to identify causes of thrombocytopenia as hyperdestructive or hypoproductive with sufficient predictive capacity, sensitivity and specificity [4, 5, 7]. In the study reported herein, three platelet indices MPV, PDW and P-LCR were analyzed for their diagnostic predictive capacity in different patients with thrombocytopenia. Consistent with the above studies [4, 5, 7], there was a significant difference in MPV between hypoproductive and ITP patients. Moreover, PDW and P-LCR had significant differences between the two patient groups and better discriminating potential.

Niethammer et al. reported that maximum of the histogram, that is the highest peak of the platelet volume distribution curve, has better efficiency than MPV in identifying thrombocytopenia caused by ITP and that resulted from decreased platelet production secondary to receiving chemotherapy [9]. MPV was able to predict the presence of bone marrow metastasis in solid tumor patients with 85 % PPV and 90 % NPV [10]. There is a growing interest in the use of platelet markers in discriminating different forms of thrombocytopenia. Monteagudo et al. measured reticulated platelets by using flow cytometry and showed this parameter has high sensitivity and specificity in identifying thrombocytopenia with increased thrombopoietic activity [11].

Studies showed immature platelet fraction (IPF) measured by Sysmex XE2100 had better sensitivity and specificity in the diagnosis of hyperdestructive thrombocytopenia like ITP and TTP [12, 13]. Though this IPF is one of the out puts from the Sysmex XE2100, such kind of automated analyzer is not available in our country for routine hematology analysis. Sysmex XT 2000i is the model currently available in our country which was also used in this study.

The most common cause of thrombocytopenia in hypoproductive patients was bone marrow suppression secondary to CML chemotherapy. This could be due to the predominance of CML as the leading type of leukemia in Ethiopia which accounts for more than 57.8 % of all the leukemia causes [14].

There was uneven distribution of sex among the study subjects, where females were almost twice than males in ITP, on the other hand males were twice in hypoproductive patients. This may be due to some epidemiological differences in the incidence and prevalence of chronic ITP which is more common in females (in particular in women of child bearing age) compared to males with ratio of 2–3 to 1 [15, 16], while CML is more common in old ages and in males than females with ratios of 1.5–1.8 to 1 [17, 18].

MPV values of 9.7 fl for the hypoproductive group and 12.4 fl for ITP patients observed in our study is much higher than those reported from UK [5], Taiwan [6] and India [19]. In all these studies, they showed that MPV was significantly different between hypoproductive and hyperdestructive patients. However, the reported mean MPV values are 8.1 fl and 9.8 fl in the UK study [5], 7.2 fl and 8.8 fl in the Taiwan [6] and 7.3 fl and 8.62 fl in the Indian study [19] in hypoproductive and hyperdestructive patients, respectively.

The first possible explanation for such differences between this and the above studies could be the kind of automated hematology analyzers that is used for enumerating the platelets. A study conducted by Kaito et al. In Japan using Sysmex-XE2100 analyzer (Kobe, Japan) reported a mean MPV of 10.2 fl in aplastic anemia and 12.2 fl in ITP patients [7], whose values are closer to our finding for ITP. A similar finding was also reported from a study conducted by Ntaios et al. who also used sysmex-XE2100 automated analyzer [4]. Ntaios et al. emphasized that the higher values of the platelet indices in their study compared to others could be due to a difference in hematology analyzers. Large or giant platelets could be excluded from the platelet count with instruments which use impedance method like coulter STKS [6] and coulter Gen-S [5] used by the above studies, but the Sysmex XT2000i and XE2100 series also employ optical fluorescence detection method [20].

The second possible reason could be an actual difference in the platelet indices among population from country to country. A study by Hong et al. in healthy Chinese adults using Sysme XT 2100 indeed confirmed variations of platelet indices between regions. The reported value of MPV for example ranged from 10.30 ± 0.80 to 12.36 ± 1.34 [21]. In another study by Maluf et al. a statistically significant difference in MPV, PDW, and P-LCR according to self-declared race/skin color was demonstrated, where mean MPV, PDW, and P-LCR values of white individuals were lower than those of individuals self-declared black or pardo (mixed skin color/brown) [22]. An earlier study by Barbara J to evaluate ethnic and sex differences in hematological profiles including platelet count and MPV reveled an MPV of 8.9 fl in Caucasians (eastern Europe), 9.1 fl in Afro-Caribbean’s and 9.4 fl in Sub-Saharan Africans (including few Ethiopians) [23]. It is evident that Africans have higher MPV as compared to the other groups. Though we have analyzed 42 healthy controls, the mean MPV was 10.3 fl which suggest that the actual size of the platelet in our study subjects could be higher.

The mean MPV of the healthy controls (10.3 fl) in the current study can identify ITP patients with 82 % sensitivity, 66 % specificity, 61.4 % positive predictive value and 85 % negative predictive value. In a similar study conducted by T. Numbenjapon et al. in healthy Thais, the mean MPV was 7.9 fl. This identified hyperdestructive thrombocytopenic patients with a sensitivity of 82.3 %, a specificity of 92.5 %, a PPV of 94.4 %, and a NPV of 77.1 % [6]. Kaito et al. analyzed the role of MPV, PDW and P-LCR in the diagnosis of ITP and reported somewhat an improved sensitivity and specificity where an MPV of >11 fl has a sensitivity of 87.2 % and a specificity of 80.0 % [7].

This variation in sensitivity, specificity and predictive values could be due to differences in the type of study participants, where some have compared only one patient group, bone marrow disease due to aplastic anemia against ITP [4, 7]. However, in our study more than 8 causes of bone marrow disease were identified. The other reason could be the predominance of chronic ITP which accounts 28 out of 33 ITP patients. These patients have relatively stable platelet count, on average 88 × 10^9^/L and a mean MPV of 11.6 fl with a range of 9.2 to 17. On the other hand hypoproductive patients have a mean MPV of 9.7 fl with arrange of 7.1 to 11.2; hence it is evident that some platelet indices values are overlapping between the two patient groups.

Supporting the above explanation in vitro studies showed that plasma auto antibodies from ITP patients not only are involved in platelet destruction, but may also contribute to the inhibition of platelet production by affecting megakaryocyte production and maturation [24, 25]. It suggests that a similar effect may occur in vivo. This may partly explain the lower platelet indices in our chronic ITP patients compared to the acute ITP cases. Despite hyper-destructive thrombocytopenia which is characterized by higher MPV, these chronic patients may have normal or even suppressed platelet production rate.

The significant negative correlation between the platelet count and the platelet indices in ITP patients seems to be mainly contributed by acute ITP patients who had a mean platelet count of 12.6x10^9^/L and mean MPV of 16.6 fl, PDW 19 fl and P-LCR of 51.5 %. This shows the platelet indices in particular MPV and P-LCR could have a better discriminating or prediction capacity for ITP where they are needed the most, during the early investigation and diagnosis of thrombocytopenic patients. Among the 33 ITP patients, 10 of them had bone marrow examination with result of no primary or secondary bone marrow disease. If not in all these 10 patients, the platelet indices could have assisted in predicting most patients to have ITP and could prevent them from undergoing this invasive procedure.

## Conclusion

In conclusion these indices in particular MPV and P-LCR can discriminate ITP from hypoproductive thrombocytopenia and they may help in avoiding or delaying ITP patients from undergoing unnecessary, invasive bone marrow aspiration or prevent undesirable platelet transfusion. The mean platelet indices are relatively higher in our study groups. Therefore, cut off values need to be established on the given laboratory setup and place for the indices to be used as a discriminating tool for thrombocytopenia. Future studies comparing impedance and impedance with optical method and inclusion of more types of hyper-destructive thrombocytopenia may enable us to use these indices for broader patient groups.

### Limitation of the study

When there is abnormal distribution of platelets or fragments of RBCs and blasts, the analyzer may not display the values of platelet indices; thus, interpreting the result from the histogram and reviewing the smear could be necessary. Relatively the smaller sample size and limited disease categories in particular in hyper destructive thrombocytopenia group may limit application of this finding in broader patient groups.

## Abbreviations

ALL, acute lymphocytic leukemia; AML, acute myelocytic/myelogenous leukemia; BM, bone marrow; CBC, complete blood count; CLL, chronic lymphocytic leukemia; CML, chronic myolegenous leukemia; DIC, disseminated intravascular coagulation; EDTA, ethylene diamine tetra-acetic acid; fL, femto liter (10^−15^ litre); Hb, hemoglobin; HIV, human immunodeficiency virus; IPF, immature platelet fraction; IPU, information processing unit; ITP, immune thrombocytopenic purpura; IVIg, intravenous Immunoglobulin; MCH, mean cell hemoglobin; MCV, mean cell volume; MPV, mean platelet volume; NPV, negative predictive value; PCT, platecrit; PDW, platelet size distribution width; P-LCR, platelet large cell ratio; PPV, positive predictive value; RDW, red cell distribution width; RNA, ribonucleic acid; ROC, receiver operating curve; RP, reticulated platelets; TTP, thrombotic thrombocytopenic purpura
